# P-1127. Compliance Matters: Reversal of Infection Reduction in Hip Arthrodesis Due to Surgical Procedure Audit Failure

**DOI:** 10.1093/ofid/ofaf695.1321

**Published:** 2026-01-11

**Authors:** Raquel B Silva, Gabrielle A R Mota, Laila G Machado, Thiago C Gontijo, Gabriel Colen, Ana Carolina Morganti, Bráulio R G M Couto

**Affiliations:** Hospital Universitário Ciências Médicas (HUCM), Belo Horizonte, Minas Gerais, Brazil; Hospital Universitário Ciências Médicas (HUCM), Belo Horizonte, Minas Gerais, Brazil; Hospital Universitário Ciências Médicas (HUCM), Belo Horizonte, Minas Gerais, Brazil; University Hospital Medical Sciences, Belo Horizonte, Minas Gerais, Brazil; Hospital Universitário Ciências Médicas (HUCM), Belo Horizonte, Minas Gerais, Brazil; Hospital Universitário Ciências Médicas (HUCM), Belo Horizonte, Minas Gerais, Brazil; AMECI – Associação Mineira de Epidemiologia e Controle de Infecções, Belo Horizonte, Minas Gerais, Brazil

## Abstract

**Background:**

In 2022, the risk of surgical site infection (SSI) after hip arthrodesis was very high (11%). Subsequently, the infection control service implemented an SSI prevention bundle composed of six measures (Fig. 1). The SSI rate dropped immediately, resulting in only one case in 2024, with an SSI rate of 0.8%. However, at the beginning of 2025, the SSI rate sharply increased to 12%, returning to the 2022 level (Table 1). The objective of our study is to evaluate how failure in adherence to the surgical site infection prevention bundle can lead to the SSI peak after hip arthrodesis.

**Figure 1:**
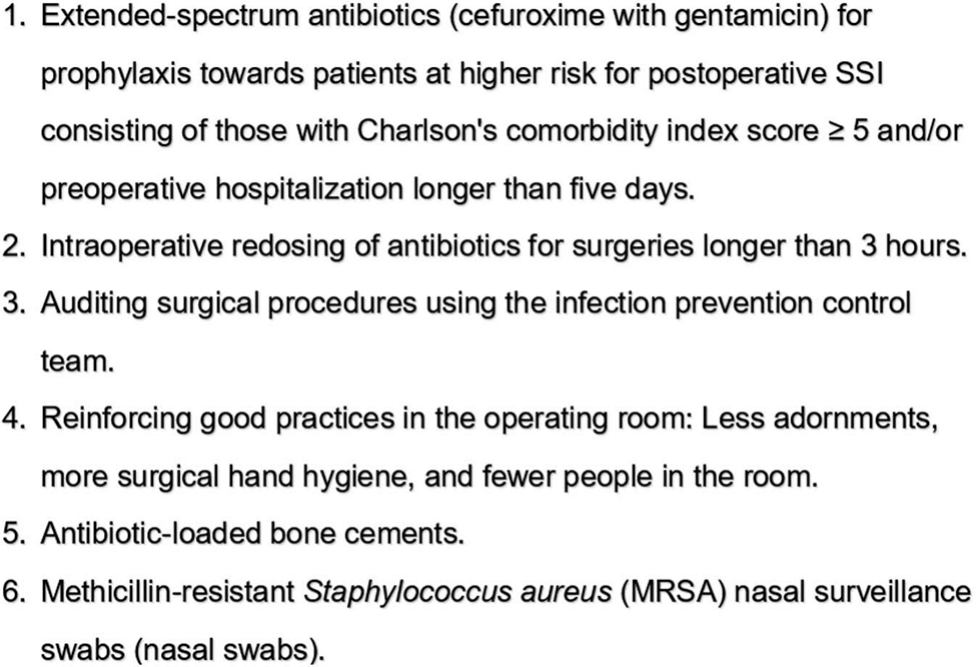
Surgical site infection prevention bundle. Surgical site infection prevention bundle.

**Table 1:**
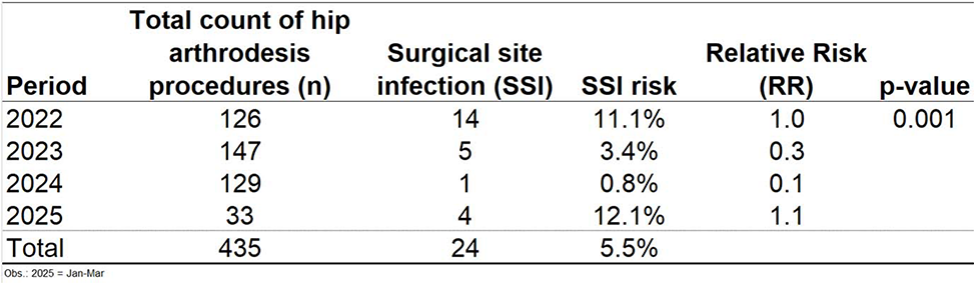
Surgical Site Infection Rate After Hip Arthrodesis: Reversal of Reduction in 2025, Returning to 2022 Levels. Surgical Site Infection Rate After Hip Arthrodesis: Reversal of Reduction in 2025, Returning to 2022 Levels.

**Methods:**

A single-center retrospective cohort study was conducted between Jan/2022-Mar/2025, involving patients undergoing hip arthrodesis. SSI was defined according to the criteria set by the CDC. Beginning in Sep/2024, we implemented a compliance assessment system to assess adherence to the surgical site infection prevention bundle items through sampling. Additionally, we assessed other preventive and risk behaviors during surgery using a questionnaire composed of 26 questions.

**Figure 2:**
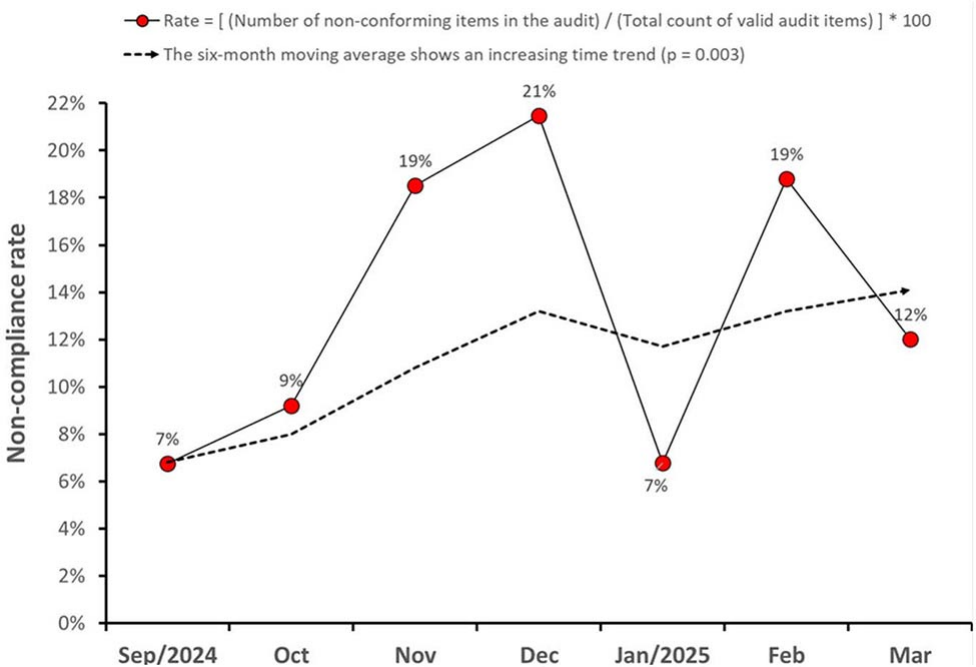
Surgical Procedure Audit Non-Compliance Increased Significantly from the end of 2024 to the beginning of 2025. We observed a significant increase in the non-compliance rate with preventive SSI measures towards the end of 2024 and the beginning of 2025

**Table 2:**
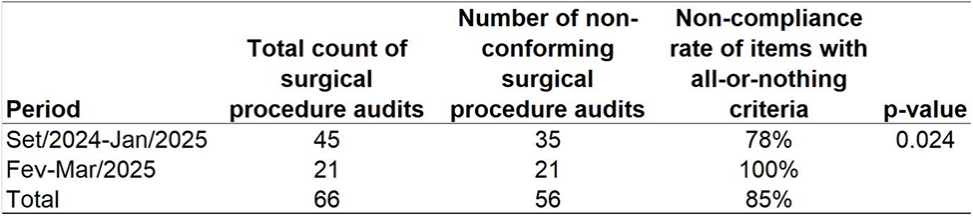
Analysis all-or-nothing approach Surgical Procedure Audit Non-Compliance: Marked Increase Observed in February-March 2025. The non-compliance rate increased from 78% between September 2024 and January 2025 to 100% in February and March 2025

**Results:**

Between Sep/2024-Mar/2025, we audited 66 surgical procedures. This sample included not only hip arthrodesis but also other surgical procedures. We observed a significant increase in the non-compliance rate with preventive SSI measures towards the end of 2024 (Fig. 2). The non-compliance rate increased from 78% between Sep/2024-Jan/2025 to 100% in Feb-Mar/2025 (Table 2). Univariate analysis of potential risk factors for SSI, comparing data from 2024 and 2025, did not identify any significant associations. The correlation between the non-compliance rate and SSI incidence was the only factor identified that could explain the observed SSI peak. Consequently, the Infection Control Service met with orthopedic and other surgeons to present these findings and emphasize the urgent need to improve adherence to preventive measures.

**Conclusion:**

Despite the inherent limitations of our study, the considerable increase in non-compliance with SSI preventive measures appears to have driven the peak in SSI rates post-hip arthrodesis. Thus, improved adherence to these measures is crucial for prevention. Our prevention strategies are currently under further evaluation to better understand these findings.

**Disclosures:**

All Authors: No reported disclosures

